# Preschool overweight and obesity in urban and rural Vietnam: differences in prevalence and associated factors

**DOI:** 10.3402/gha.v8.28615

**Published:** 2015-10-08

**Authors:** Loan Minh Do, Toan Khanh Tran, Bo Eriksson, Max Petzold, Chuc T. K. Nguyen, Henry Ascher

**Affiliations:** 1 Outpatient Department, National Hospital of Paediatrics, Hanoi, Vietnam; 2 Section for Epidemiology and Social Medicine (EPSO), Department of Public Health and Community Medicine, Institute of Medicine, Sahlgrenska Academy, University of Gothenburg, Gothenburg, Sweden; 3 Family Medicine Department, Hanoi Medical University, Hanoi, Vietnam; 4 Health Metrics, Department of Public Health and Community Medicine, Institute of Medicine, Sahlgrenska Academy, University of Gothenburg, Gothenburg, Sweden; 5 School of Public Health, Faculty of Health Sciences, University of the Witwatersrand, Johannesburg, South Africa; 6 Angered Hospital, Gothenburg, Sweden

**Keywords:** overweight, obesity, preschool children, Vietnam

## Abstract

**Background:**

Childhood obesity may soon be an equally important health threat as undernutrition and infectious diseases. Accurate information about prevalence and risk factors of obesity in children is important for the design of prevention.

**Objective:**

The aim of this study was to estimate prevalence of overweight and obesity for preschool children in two Vietnamese areas, one urban and one rural, and to identify risk factors.

**Design:**

A cross-sectional study was conducted in urban Dong Da and rural Ba Vi districts, Hanoi, Vietnam. Totally, 2,677 children, 1,364 urban and 1,313 rural, were weighed and measured. Caregivers were interviewed. Background information about children and families was obtained from regular household surveys.

**Results:**

The prevalence of overweight and obesity combined were 21.1% (95% CI 18.9–23.3) in the urban area and 7.6% (95% CI 6.2–9.2) in the rural. Multiple logistic regression revealed that at the individual level, in both sites, the risk increased with increased child age. The identified urban risk factors were being a boy, consuming large amounts of food, eating fast, and indoor activity less than 2 hours per day. The rural risk factors were frequent consumption of fatty food. At the family level, significant association was found in rural areas with frequent watching of food advertisements on television.

**Conclusions:**

Overweight and obesity are emerging problems in Vietnam, particularly in the urban context. Prevention programs should focus on education about healthy eating habits at early preschool age and need to be tailored separately for urban and rural areas since the risk factors differ. Non-healthy food advertisement needs to be restricted.

The World Health Organization (WHO) predicts that childhood obesity may soon be an equally important health threat as undernutrition and infectious diseases (1). Globally, the prevalence of overweight and obesity among preschool children increased from 4.2% in 1990 to 6.7% in 2010 (2). This development increasingly extends into low- and middle-income countries causing a public health problem. Genetic susceptibility, environment, socioeconomy, and individual lifestyle are factors believed to play important roles for the development of obesity (3, 4), although their importance may differ between countries.

Vietnam is a middle-income country in rapid transition from a socialist economy to a market driven one. Between 2001 and 2010, the average economic growth was 7.3% per year (5). During the transitional period (1999–2009), the prevalence of childhood underweight has been reduced at an average rate of 1.3% per year (6). At the same time, the prevalence of overweight and obesity among children under 5 years of age have increased (6). Like in many countries, the gaps in economic and social conditions have widened in Vietnam. The mean monthly income per capita in the urban areas was equal to $140 in 2012, approximately double that in the rural areas (7).

There is a paucity of research examining to what extent Vietnamese preschool children have developed overweight and obesity and the effects of the rapid socioeconomic changes on development of childhood obesity. The aim of this study was to estimate the prevalence of overweight and obesity among preschool children in one urban and one rural area of Hanoi, Vietnam, and to study associations with individual and family factors. The aim was also to establish baseline information for follow-up of the participating children.

## Methods

**Fig. 1:**
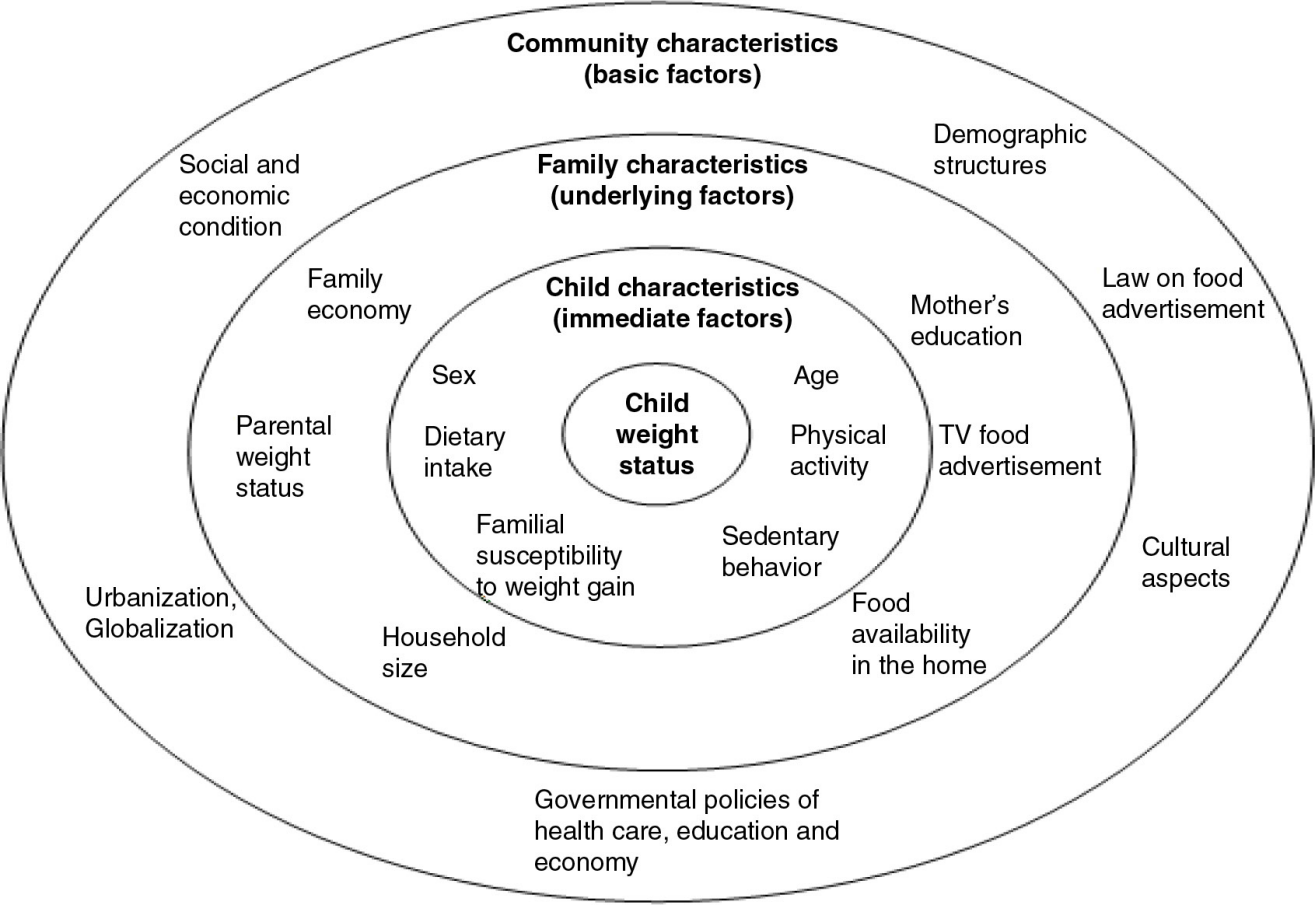
Conceptual framework for the study.

The conceptual framework for overweight and obesity used for the study (Fig. 1) was adapted from a model given by Davison and Birch (8). The factors included were conceptualized at the levels of individual, family, and community. The basic assumption is that factors in the outer circles affect those in the inner circles. However, they are correlated and interact with each other to influence child weight status. For instance, diet, physical activity, and sedentary lifestyle characteristics of children directly affect children's weight but are in turn shaped by parenting styles and the family environment in which children are embedded. Community characteristics indirectly influencing the development of child weight in general and overweight in particular are considered to be the most important basic factors. Changes in community or society level with respect to socioeconomic conditions, culture, as well as governmental policies (health care, education, agriculture, etc.) impact both the individual and family levels.

The child and family characteristics of the model were all considered in the study using empirical information and their roles in the two community contexts, urban and rural, were discussed.

## Study sites and design

A cross-sectional study was conducted in 2013 in two Health and Demographic Surveillance Sites (HDSS) located in Hanoi, Vietnam. The rural site, FilaBavi, has a cohort of about 11,000 households and 51,000 persons, originally randomly selected in 1999. This cohort has then served as a panel for a manifold of studies over the years. It represents the different area types in the district, riverside, lowland, and mountainous areas (9). The urban site, DodaLab, was formed in 2007 by three communes strategically selected to represent slightly different social and economic conditions in Dong Da district. At that time, the DodaLab cohort had 11,000 households and 38,000 people (10). Several studies have been conducted in DodaLab most often in parallel with FilaBavi.

## Subjects

A sample size of at least 1,200 children per site was derived from a requirement that a 95% confidence interval for the estimated prevalence of overweight or obesity should have a maximum length of 5% units assuming the maximum prevalence to be 20%.

All 2,842 children born from January 1, 2007, to December 31, 2009, in some strategically selected communes, 1,482 in the urban site and 1,360 in the rural site, were included in the study. Weight and height information was obtained from 1,364 children in the urban site and from 1,313 in the rural site, making the overall participation percentage 94.2%.

## Measurement and data collection

Information about demographic and socioeconomic status (SES) of the mothers and fathers was obtained from the HDSS databases as recorded in 2012. Child eating habits and lifestyle data as well as other characteristics of the family were collected by interviewing parents or caregivers using structured questionnaires specifically designed for this study. The weight and height of children as well as their parents were obtained by well-trained staff working in pairs. Digital Tanita scales and mobile measurement instruments were used to measure the children in their homes.

*Overweight and obesity*: They were defined using WHO standards. For children under 5 years, BMI-for-age above +2 but below or equal to +3 z-scores were classified as being overweight. Obesity was defined as z-score above +3 (11). For children aged between 5 and 6 years, overweight was classified as BMI-for-age above +1 but below or equal to +2 z-score. Obesity was defined as z-score above +2 (12). For parents, overweight was defined as BMI between 25 and 30 kg/m^2^ and obesity as BMI above 30 kg/m^2^.

*Amount of food*: The portion size of food that a child eats in each main meal was compared with other children in the same age group and classified by the parents at one of three levels: ‘less than’, ‘the same’, and ‘more than’.

*Food consumption*: Frequencies of different food intakes were categorized by the parents into six levels: 1) never or less than once a week, 2) 1–3 times/week, 3) 4–6 times/week, 4) 1 time/day, 5) 2 times/day, and 6) 3 times or more/day. Fatty foods included fatty meat and butter. Fried foods were vegetable, meat, fish, and eggs that were fried.

*Fast eating:* It was defined by the parents comparing with other children in the same age group. The question asked was ‘Do you think your child eats “fast”, “normal”, or “slow” during daily meals?’.

*Irregular snacks*: They were defined as portions of food normally smaller than a regular meal that children eat at any time. Three categories were used: ‘never or rarely’, ‘sometimes’, and ‘often’.

*Outdoor physical activity*: It was estimated as the daily time, in hours, for walking, running, jumping, playing in the yard or street around the house or at a playground.

*Indoor physical activity*: It was estimated as the daily time, in hours, for playing with peers, toys, going up and down stairs, or doing house work.

*Sedentary time*: It was defined as the estimated daily time, in hours, for watching television and playing computer game.

*Family economy:* The first factor estimated in a principal component analysis of the variables indicating assets available in the households was used as an indicator of family economy. In the analysis, the variable was bracketed into terciles (13).

*Mother's education*: The level of education was categorized into three categories: secondary school or less, high school, and higher than high school.

*Household size*: The number of people living in the household.

*Watching food advertisement*: The possible answers were ‘no’, ‘sometimes’, and ‘often’ to the questions if parents watched TV food advertisements. To analyze, ‘sometimes’ and ‘often’ were combined to a category ‘yes’.

*Snack availability*: The possible answers were ‘no’, ‘yes, sometimes’, and ‘yes, often’.

## Statistical analysis

Conventional statistical methods were used to summarize and describe data in tables and graphs. Simple logistic regressions were used to study the crude statistical associations between the binary dependent variables indicating overweight and obesity and background variables. Thus, a crude odds ratio was estimated for each independent variable.

The correlations between the independent variables were studied using Spearman rank correlation coefficients.

Multiple logistic regression models, one for the rural area and another for the urban area, were used to further explore the associations and to identify and adjust for confounding. The dependent variable in these models was a binary variable indicating overweight or obesity, yes or no. The independent variables were all variables excluding parents’ weight status since many values were missing. A joint model with the above covariates and an urban–rural indicator was tried but abandoned since the two areas are structurally too different for a meaningful simultaneous interpretation.

The goodness of fit of the models was explored using the Hosmer–Lemeshow test. Predictive value was studied using various measures such as the McFadden *R*^2^ and the Tjur's coefficient of discrimination (14). Statements of statistical significance were based on the comparison of confidence intervals or *p*-values. For comparisons of two groups, for example, urban and rural, Student's *t*-test or Wilcoxon rank sum test was used as appropriate. The software used for description and analysis was Stata version 14.

## Ethical considerations

The field sites in FilaBavi and DodaLab have ethical approvals from the Ministry of Health of Vietnam as well as from the Scientific and Ethical Committee of Hanoi Medical University. Permissions have also been given by Dong Da and Ba Vi district authorities. Oral consent from all caregivers of children participating in the studies was obtained.

## Results

### Characteristics of the study populations

Table 1 shows characteristics for the distributions of child and family variables used in the study of associations with overweight or obesity, by site. Compared with rural children, urban children had less outdoor activity and more sedentary time. Urban families had more assets and reported higher income than the rural families. The urban parents also had higher education. The mean BMI of mothers and fathers in the urban site were 21.5 and 22.5, respectively, which was higher than in the rural area with 20.3 and 21.1, respectively.

**Table 1:** Descriptive characteristics of child, parent, and household variables used in the study

Variables	Urban (*n*=1,364)	Rural (*n*=1,313)
Child age, years (mean)	4.3	4.0
Boys (%)	53.1	53.7
Girls (%)	46.9	46.3
Child BMI (mean±SD)	15.9±2.44^b^	15.3±1.86
Indoor activity, minutes (median)	162.9^b^	120.0
Outdoor activity, minutes (median)	103.9^b^	154.3
TV viewing, minutes (median)	60.0^b^	38.6
Computer game, minutes (median)	0.0	0.0
Number of family assets^a^ (median)	11^b^	5
Mothers with secondary school or less (%)	6.8^b^	57.6
Mothers with high school education (%)	32.3^b^	28.6
Mothers with education higher than high school (%)	60.9^b^	13.8
Number of people in the household (median)	4	4
Mother's BMI (mean±SD)	21.5±2.27^b^	20.3±2.01
Father's BMI (mean±SD)	22.5±2.28^b^	21.1±2.09

a Replaced by the first factor from principal component analysis in subsequent regressions;

b Statistically significant difference between the urban and rural sites (*p*<0.001). Student's *t*-test for variables with symmetric distribution, Wilcoxon rank sum test for others.

The child and family variables were to varying extent correlated. However, the estimated correlations were not very strong and mainly smaller than 0.30. The only case of substantial collinearity found when testing the models using the variance inflation factor (VIF) was between the variables for mother and father viewing TV advertisements. The strongest correlations were seen between mother's and father's viewing of TV food commercials and unhealthy food consumption as well as sedentary behavior in the urban area. This was not seen for the rural area. The correlations between mother's education and household assets were 0.32 and 0.35 in the urban and rural areas, respectively. There were generally stronger correlations in the urban area than in the rural area. Overall, there were quite several correlation estimates that were statistically significantly different from zero. However, given the sample sizes above 1,300 in each area, the limit for being statistically significant at 5% is about 0.06.

### Prevalence of overweight and obesity

Table 2 shows the estimated prevalence of child overweight and obesity. The urban prevalence was statistically significantly higher than those for the rural area. Boys were more likely than girls to be obese in the urban area.

**Table 2:** Estimated prevalence of overweight and obesity as defined using WHO standard

	Number of children	Overweight % (95% CI)	Obesity % (95% CI)	Overweight or obesity % (95% CI)
All children	2,677	8.4 (7.3–9.5)	6.1 (5.2–7.1)	14.5 (13.1–15.8)
Urban total	1,364	12.2 (10.5–14.0)	8.9 (7.4–10.5)	21.1 (18.9–23.3)
Urban boys	725	13.2 (10.9–15.9)	11.9 (9.6–11.4)	25.1 (22.0–28.4)
Urban girls	639	11.0 (8.6–13.6)	5.5 (3.8–7.5)	16.4 (13.6–19.5)
Rural total	1,313	4.4 (3.4–5.7)	3.2 (2.3–4.3)	7.6 (6.2–9.2)
Rural boys	705	4.8 (3.4–6.7)	3.3 (2.1–4.9)	8.1 (6.2–10.3)
Rural girls	608	4.0 (2.5–5.8)	3.1 (1.9–4.8)	7.1 (5.2–9.40)

### Crude associations between combined overweight and obesity and factors at individual and family levels

Tables 3 and 4 show the crude associations between combined overweight and obesity and the variables given in the child and family spheres of the conceptual framework. At the individual level, in both sites, there was statistically significant association between child overweight including obesity and age. Significant associations were found for being a boy, large amounts of food, fast eating, and indoor activity in the urban area, and for frequent consumption of fatty, fried food; irregular snacks; and indoor activity and outdoor activity in the rural area.

**Table 3:** Results of simple logistic regression of combined overweight and obesity on individual child factors

		Urban population (*n*=1,364)				Rural population (*n*=1,313)			
			
Variables		*n*	Prevalence (%)	OR	95% CI (*p*-value)	*n*	Prevalence (%)	OR	95% CI (*p*-value)
Sex	Female	639	16.4	Ref		608	7.1	Ref	
	Male	725	25.1	1.70	1.30–2.23	705	8.1	1.16	0.77–1.74
					(<0.001)				(0.49)
Age	3	323	10.8	Ref		442	5.4	Ref	
	4	455	14.5	1.40	0.90–2.16	420	4.3	0.79	0.42–1.46
	5	480	34.0	4.23	2.84–6.30	406	13.6	2.73	1.66–4.50
	6	106	21.7	2.28	1.28–4.07	45	6.7	1.24	0.36–4.31
					(<0.001)				(<0.001)
Amount of food	Normal	1.116	21.0	Ref		841	8.4	Ref	
	Large	76	43.4	2.89	1.80–4.66	84	7.1	0.82	0.35–1.96
					(<0.001)				(0.28)
Fatty food	<1 time/week	407	21.1	Ref		945	6.6	Ref	
	1–6 times/week	694	22.3	1.07	0.80–1.45	298	5.0	0.75	0.42–1.35
	≥7 times/week	233	18.0	0.82	0.54–1.24	63	34.9	7.64	4.29–13.63
					(0.37)				(<0.001)
Fried food	<7 times/week	824	19.9	Ref		869	6.2	Ref	
	≥7 times/week	531	22.6	1.18	0.90–1.53	440	10.2	1.72	1.14–2.60
					(0.24)				(0.01)
Irregular snack	No	223	23.8	Ref		193	3.1	Ref	
	Sometimes	796	20.1	0.81	0.57–1.15	577	6.1	2.01	0.83–4.86
	Often	345	21.5	0.88	0.59–1.31	542	10.9	3.81	1.62–8.97
					(0.49)				(<0.001)
Eating speed	Normal	877	20.8	Ref		653	10.1	Ref	
	Fast	168	36.9	2.22	1.56–3.16	200	4.5	0.42	0.21–0.86
					(<0.001)				(0.01)
Outdoor activity	≥2 hours/day	563	18.7	Ref		1.066	6.7	Ref	
	<2 hours/day	801	22.7	1.28	0.98–1.68	247	11.8	1.87	1.18–2.94
					(0.07)				(0.01)
Indoor activity	≥2 hours/day	989	19.2	Ref		742	5.4	Ref	
	<2 hours/day	375	25.9	1.47	1.11–1.94	571	10.5	2.06	1.36–3.12
					(0.01)				(<0.001)
Sedentary behavior	<2 hours/day	1.091	21.8	Ref		1.218	8.0	Ref	
	≥2 hours/day	272	18.0	0.79	0.56–1.11	94	3.2	0.38	0.12–1.23
					(0.16)				(0.06)

The *p*-values refers to the overall test comparing all categories. Ref: reference group.

**Table 4:** Results of simple logistic regression of combined overweight and obesity on family factors

		Urban population (*n*=1,364)				Rural population (*n*=1,313)			
			
Variables		*n*	Prevalence (%)	OR	95% CI (*p*-value)	*n*	Prevalence (%)	OR	95% CI (*p*-value)
Family economy	Group 1	83	19.3	Ref		983	7.02	Ref	
	Group 2	476	18.7	0.96	0.53–1.74	305	9.2	1.34	0.85–2.12
	Group 3	803	22.5	1.22	0.69–2.15	25	12.0	1.81	0.53–6.19
					(0.24)				(0.35)
Mother's education	Secondary or less	89	19.1	Ref		738	7.5	Ref	
	High school	421	20.2	1.07	0.60–1.91	366	7.5	1.03	0.64–1.65
	Higher than high	793	22.1	1.20	0.69–2.09	177	9.6	1.32	0.75–2.33
	school				(0.65)				(0.64)
Household size	≤6 people	1.276	20.8	Ref		966	8.5	Ref	
	>6 people	86	24.4	1.23	0.74–2.05	314	4.8	0.54	0.31–0.95
					(0.43)				(0.02)
Watching food advertisements on TV by mother	No	622	21.1	Ref		728	4.1	Ref	
	Yes	734	21.1	1.00	0.77–1.30	561	12.5	3.32	2.13–5.17
					(0.98)				(<0.001)
Watching food advertisements on TV by father	No	663	21.7	Ref		782	6.3	Ref	
	Yes	668	20.5	0.93	0.71–1.21	691	10.4	1.73	1.15–2.61
					(0.59)				(0.01)
Snack availability	No	478	19.5	Ref		772	6.0	Ref	
	Yes	886	21.9	1.16	0.88–1.53	540	10.0	1.75	1.16–2.64
					(0.29)				(0.01)
Parents’ weight status	Non-obese	1.002	20.6	Ref		1.032	8.0	Ref	
	At least one obese parents	219	26.0	1.38	1.02–1.87 (0.04)	54	16.7	2.32	1.17–4.59 (0.03)

The *p*-values refers to the overall test comparing all categories. Ref: reference group.

At the family level (Table 4), the risk of being overweight or obese was increased among children with obese parents compared with children of non-obese parents in both sites. Factors significantly associated only in the rural area were watching food advertisements on TV, household size, and availability of snacks. The odds of child overweight and obesity increased in children with mothers having higher education although not statistically significant.

### Multiple logistic models

Table 5 shows the results for the two separate urban and rural areas multiple logistic regression models. In the urban area, all variables that were statistically significant in the simple models remained statistically significant. In the rural area, age, fatty food, and mothers watching food advertisements on TV were statistically significantly associated.

**Table 5:** Results of multiple logistic regression analysis of combined overweight and obesity

		Urban		Rural	
			
Variables		OR	95% CI	OR	95% CI
Sex	Female	Ref		Ref	
	Male	1.87	1.38–2.52	1.20	0.76–1.90
Age	3	Ref		Ref	
	4	1.56	0.97–2.50	0.77	0.39–1.50
	5	4.84	3.13–7.48	2.57	1.47–4.51
	6	2.30	1.21–4.38	0.83	0.21–3.26
Amount of food	Normal	Ref		Ref	
	Large	2.25	1.21–4.16	1.43	0.48–4.23
Fatty food	<1 time/week	Ref		Ref	
	1–6 times/week	1.10	0.78–1.54	0.75	0.40–1.42
	≥7 times/week	0.86	0.54–1.37	4.28	2.09–8.73
Fried food	<7 times/week	Ref		Ref	
	≥7 times/week	1.14	0.81–1.60	1.19	0.70–2.00
Irregular snack	No	Ref		Ref	
	Sometimes	0.75	0.50–1.15	1.58	0.62–4.05
	Often	0.83	0.52–1.33	2.28	0.89–5.84
Eating speed	Normal	Ref		Ref	
	Fast	1.84	1.16–2.92	0.50	0.20–1.22
Outdoor activity	≥2 hours	Ref		Ref	
	<2 hours	1.29	0.94–1.75	1.24	0.73–2.13
Indoor activity	≥2 hours	Ref		Ref	
	<2 hours	1.44	1.01–2.05	1.40	0.85–2.32
Sedentary behavior	<2 hours	Ref		Ref	
	≥2 hours	0.75	0.50–1.12	0.74	0.21–2.57
Family economy	Group 1	Ref		Ref	
	Group 2	1.03	0.50–2.13	1.19	0.69–2.05
	Group 3	1.16	0.56–2.41	1.10	0.25–4.84
Mother's education	Secondary or less	Ref		Ref	
	High school	1.27	0.65–2.48	0.86	0.50–1.47
	Higher than high school	1.45	0.74–2.84	1.11	0.55–2.23
Household size	≤6 people	Ref		Ref	
	>6 people	1.12	0.64–1.97	0.67	0.36–1.23
Watching food advertisements on TV by mother	No	Ref		Ref	
	Yes	1.20	0.71–2.01	3.24	1.67–6.28
Watching food advertisements on TV by father	No	Ref		Ref	
	Yes	0.69	0.41–1.16	0.73	0.40–1.32
Snack availability	No	Ref		Ref	
	Yes	1.15	0.83–1.60	0.80	0.46–1.38

Ref: reference group.

### Goodness of fit

Applying the Hosmer–Lemeshow test for goodness of fit to the logistic multiple regression model did not result in *p*-values small enough to reject the null hypothesis that the model used was appropriate. A well-known problem with the test is that the choice of number of groups can be crucial for the outcome. We therefore tried a series of different groupings but no small *p*-value of the Hosmer–Lemeshow chi-square was observed.

### Predictive value

The estimates of predicted value (logistic regression ‘*R*^2^’) were generally rather small. Regardless of the method used, they were less than 15% and systematically lower for the rural area.

## Discussion

The combined prevalence of overweight and obesity for rural and urban areas taken together in the present study, that is, 14.5%, was higher than the 5.6% in a national Vietnamese nutrition survey in 2010 (6). The prevalence in the urban area (21.1%) was considerably higher than in the rural area (7.6%). A study in urban Ho Chi Minh City 2005 using the International Obesity Task Force (IOTF) cutoff points showed 36.8% overweight and obesity among preschool children (15). Applying the same cutoff points to our study, the corresponding estimate was 22.6% in the Hanoi urban area. There are, however, several important differences between the two cities. Ho Chi Minh City is a business-oriented city in the south of Vietnam, which historically has had earlier contacts with Western, typically US, lifestyles and its risks for overweight and obesity. Hanoi, being the capital and administrative center in the north, experienced such influences much later. The same tendency of higher prevalence of overweight and obesity in cities having high SES has also been observed in other Asian countries, such as Thailand, China, and Malaysia, and in other developing countries (16–18). This contrasts to results from some high-income countries where obesity among children is more common in areas with low SES (19, 20).

Vietnam's economy has grown relatively fast recently. The Vietnamese GDP per capita nearly tripled between 2001 and 2010, from USD 413 to USD 1,169 (5). This may have led to improved economic conditions for persons and households but also to changes in lifestyle such as the introduction of negative eating habits and increased sedentary behavior. A survey in 2010 showed that the proportion of energy intake from fat was 8.4% in 1990 and more than double, that is, 17.6%, in 2010 (6). These changes could increase the risk for development of obesity in general and particularly in children.

Our findings indicate that some child characteristics (sex, age, eating habit, physical activity) as well as some family characteristics (parental exposure to advertisement, obese parents) were statistically associated with overweight and obesity in children. It should be kept in mind that all associations will vary between different contexts. The two sites, the urban and the rural, are likely to be different in most of the factors at the community level of the conceptual framework we used. For example, it is obvious that social and economic characteristics differ quite clearly. There are demographic differences, for example, more children per family in the rural area. Health and health care utilization patterns are different. In the ongoing rapid urbanization, one area is losing inhabitants, and thereby resources, whereas the other gains inhabitants and resources. The culture is certainly different in the urban area compared with the rural area. When used in policy work, the associations concluded above must, therefore, be seen as hints to be discussed specifically in the particular contexts rather than universal truths.

In the present study, our finding that boys were more likely to be obese than girls, especially in the urban area, is consistent with recent studies done in Vietnam (21, 22). According to the Vietnamese culture, boys play a more important role than girls in families and communities. Men are responsible for the family economy, taking care of aging parents, and carrying on the family name for future generations. Boys might receive more attention already early in life, also with respect to feeding. More ‘generous’ practices that are not necessarily positive could be established and could become one of the explanations for the higher prevalence in boys.

We found that the estimated prevalence of overweight and obesity among children aged 3–6 years increased significantly with age, with a peak at age 5 years in both boys and girls. One of the critical periods mentioned in literature for the development of obesity are the ages from 4 to 7 years (1). At these ages, BMI begins to increase rapidly following a rise in infancy and subsequent decline. An earlier adipose rebound increases the risk of later obesity (23). A study in China indicated that the age of adiposity rebound was 4 years in boys and 3 years in girls (24), and the same was seen in a study in Iran (25). In the present cross-sectional study, a cohort effect could to some extent be contributing to the increasing trend. A follow-up of the children in the present cross-sectional study may shed more light on this and is essential since the most sensitive ages must be considered when designing prevention programs for obesity.

Food consumption, both quality and quantity, is an important factor influencing weight status. The diet containing food that is high in fat will easily result in excessive energy intake which is stored as fat in the body and can lead to obesity (26). There is also supporting evidence that large portions of food along with high-energy density could contribute to an increased energy intake and subsequent weight gain (27). The results from our study support this theory. In the urban site, a large amount of food consumed by children was associated with increased odds for overweight and obesity. In contrast, the type of food intake, such as fatty food, was associated with obesity among rural children. The existence of these associations suggests that education on healthy eating habits is necessary to control overweight.

Physical activity is a crucial factor for energy expenditure. Some studies have indicated a protective role of physical activity against weight gain (24, 28). In this study, we found that more than 2 hours of physical activity, outdoors or indoors, was associated with reduction of the odds for overweight and obesity although it lost statistical significance in the multiple logistic model in the rural area. The rural children were more physically active and had less sedentary time than the urban children. The urban areas in Vietnam often lack spaces where children can play outdoors. This can be a barrier against being physical active.

Obesity is a disorder caused by a combination of genetic and environmental factors (29, 30). Individuals with a genetic susceptibility could have an increased risk for development of obesity when exposed to an adverse environment. Convincing evidence supports that there is an increased risk of being obese for children having obese parents (19, 24). The findings from our study were consistent with those results. Similarity in food preference between children and parents, possibly reflecting genetic similarities in taste perception, has been reported (8). Obese parents can influence the child's weight status not only through genetic similarity but also by their parenting style.

At the family level, economic status was not found to be associated with overweight and obesity although we observed higher prevalence of overweight among children living in the wealthiest families compared with the poorest families. A previous study in Ho Chi Minh City on preschool children (15) which had the same finding implied that economic factors may not directly impact development of overweight but indirectly influence weight status through its effect on dietary intake and physical activity pattern. Higher income could help to control weight gain by enabling family to buy more healthy, but also more expensive food. On the contrary, higher income could lead to increased risk for overweight by giving opportunities to be inactive, for example, through television viewing. These conflicting effects could be a reason for the lack of statistically significant association between family economy and overweight.

The possible effect of food advertisement, the ethics of the advertisers, and the perceptions of the audience has been discussed much in Vietnam, both by the general public and the scientific community. The food industry strives for maximal profit and present positive descriptions of their products, leading to higher consumption. In this study, watching food advertisements was more strongly associated to overweight in the rural area than in the urban. Hypothetically, this could be ascribed to different educational levels.

A cross-sectional study such as the present one can examine correlations but has a limited ability to make conclusions about causality. However, the present study was part of a long-term project. A 3-year follow-up of the same children is planned to evaluate the development of overweight and obesity.

The information about child eating habits in the study was obtained through the judgments by parents or caregivers. Several ways for obtaining such information have been proposed, all with different limitations and drawbacks. An ideal might be actually to observe the child's eating which is hardly feasible in large population studies. Observations might be made for limited groups but we should still not know if the observed eating is the same as when the child is not observed.

To combine the urban and rural areas in one model with an area type indicator variable should, due to the large discrepancies between them, have necessitated the use of several interaction variables. This would lead to considerable problems in the interpretation of the results.

## Conclusions

Overweight and obesity are emerging problems in Vietnam, particularly in the urban context. At the child level, sex, age, and eating habits were found to be immediate associated factors. At the family level, parental exposure to food advertisements was an underlying associated factor. The patterns and strengths of association differed between the urban and rural areas. Community education programs on healthy eating habits are necessary for parents or caregivers and children already at early preschool age and need to be, to some extent, differently tailored between urban and rural areas. Commercial food advertisements for unhealthy food products, for example, on TV, must be counteracted and need to be guided by laws on food advertisements.
